# Monitored anesthesia care and asleep-awake-asleep techniques combined with multiple monitoring for resection of gliomas in eloquent brain areas: a retrospective analysis of 225 patients

**DOI:** 10.1186/s41016-022-00311-2

**Published:** 2022-12-30

**Authors:** San-Zhong Li, Ning Su, Shuang Wu, Xiao-Wei Fei, Xin He, Jiu-Xiang Zhang, Xiao-Hui Wang, Hao-Peng Zhang, Xiao-Guang Bai, Guang Cheng, Zhou Fei

**Affiliations:** 1 Department of Neurosurgery, Xijing Hospital, Fourth Military Medical University, Xi’an, 710032 China; 2 Department of Radiotherapy, Xijing Hospital, Fourth Military Medical University, Xi’an, 710032 China; 3 Department of Anesthesiology, Xijing Hospital, Fourth Military Medical University, Xi’an, 710032 China

**Keywords:** Monitored anesthesia care (MAC), Asleep-awake-asleep (AAA), Retrospective analysis, Eloquent areas, Gliomas

## Abstract

**Background:**

Awake craniotomy (AC) has become gold standard in surgical resection of gliomas located in eloquent areas. The conscious sedation techniques in AC include both monitored anesthesia care (MAC) and asleep-awake-asleep (AAA). The choice of optimal anesthetic method depends on the preferences of the surgical team (mainly anesthesiologist and neurosurgeon). The aim of this study was to compare the difference in physiological and blood gas data, dosage of different drugs, the probability of switching to endotracheal intubation, and extent of tumor resection and dysfunction after operation between AAA and MAC anesthetic management for resection of gliomas in eloquent brain areas.

**Methods:**

Two-hundred and twenty-five patients with super-tentorial tumor located in eloquent areas underwent AC from 2009 to 2021 in Xijing Hospital. Forty-one patients underwent AAA technique, and the rest one-hundred eighty-four patients underwent MAC technique. Anesthetic management, dosage of different drugs, intraoperative complications, postoperative outcomes, adverse events, extent of resection and motor, and sensory and language dysfunction after operation were compared between MAC and AAA.

**Result:**

There was no significant difference in gender, KPS score, MMSE score, glioma grade, type, and growth site between the patients in the two groups, except the older age of patients in MAC group than that in AAA group. During the whole process of operation, there were greater pulse pressure difference (*P* = 0.046), shorter operation time (*P* = 0.039), less dosage of remifentanil (*P* = 0.000), more dosage of dexmedetomidine (*P* = 0.013), more use of antiemetics (81%, *P* = 0.0067), lower use of vasoactive agent (45.1%, *P* = 0.010), and lower probability of conversion to general anesthesia (GA, *P* = 0.027) in MAC group than that in AAA group. Blood gas analysis showed that PetCO2 (*P* = 0.000), Glu concentration (*P* = 0.000), and PaCO2 (*P* = 0.000) were higher, but SPO2 (*P* = 0.002) and PaO2 (*P* = 0.000) were lower in MAC group than that in AAA group. In the postoperative recovery stage, compared with that of AAA group, the probability of dysfunction in MAC group at 1, 3, 5, and 7 days after operation was lower, which were 27.8% vs 53.6% (*P* = 0.003), 31% vs 68.3% (*P* = 0.000), 28.8% vs 63.4% (*P* = 0.000), and 25.6% vs 58.5% (*P* = 0.000), respectively.

**Conclusion:**

Compared with AAA, it seems that MAC has more advantages in the management for resection of gliomas in eloquent brain areas, and MAC combined with multiple monitoring such as cerebral cortical mapping, neuronavigation, and ultrasonic detection is worthy of popularization for the resection of gliomas in eloquent brain areas.

**Supplementary Information:**

The online version contains supplementary material available at 10.1186/s41016-022-00311-2.

## Background

More than 100 years ago, awake craniotomy (AC) was used for intractable epileptic surgery [1], but since 1980s, more and more evidence has demonstrated that AC combined with intraoperative functional mapping play an important role in maximum tumor resection and maximum protection of neurological function [2–4], especially for resection of gliomas located in eloquent brain areas such as language, motor, and supplementary motor areas [2, 5]. The major challenge of AC is the maintenance of stable sedation and analgesic status, ideal hemodynamics, and normal respiratory function at the same time. Normally, there are two main anesthesia techniques for AC including the asleep-awake-asleep (AAA) and the monitored anesthesia care (MAC) techniques, and it is still debated now which one is better for resection of tumors in eloquent brain areas. A European survey [6] from 20 specialized medical centers in 11 countries indicated that although there were an equivalent proportion of centers using AAA or MAC anesthetic approach, currently no absolute consensus has been made about the best anesthetic management to carry out this kind of surgery. In our study, a retrospective analysis of 225 patients was made mainly focusing on the differences in changes of physiological and blood gas data, dosage of different drugs, the probability of switching to endotracheal intubation, extent of tumor resection, and dysfunction after operation between AAA and MAC anesthetic management for resection of gliomas in eloquent brain areas.

## Methods

### Study design and setting

In this study, we retrospectively analyzed surgical cases performed between June 2009 and June 2021 in Xijing Hospital. This study was in compliance with the Declaration of Helsinki and approved by the Research Ethics Committee of Xijing Hospital. Informed consent was signed by the patients or their legal representatives.

### Patients

This study included 225 patients who underwent surgical resection of supratentorial gliomas located within or adjacent to functional areas. The diagnoses were identified by two neurosurgeons and two radiologists. These patients had AC operations in which 41 are from the AAA group and 184 from the MCA group (Sup. Figure 1).

In our study, patients under the age of 18 years and with a Karnofsky performance status (KPS) score ≤ 60 were excluded. Patients with an American Society of Anesthesiologists (ASA) physical status classification of ≥ 4 and patients diagnosed as other malignant tumors were also excluded (Table 1).

**Table 1:** Patient characteristics

Preoperative characteristics	MAC^a^(%)	AAA^b^(%)	*p*-value
Age, years	48.26 ± 13.30	39.41 ± 12.27	0.000
Sex			
Male	95 (51.6)	21 (51.2)	0.962
Female	89 (48.4)	20 (48.8)	
KPS^c^ score			
80–90	71 (38.6)	15 (36.6)	0.811
100	113 (61.4)	26 (63.4)	
MMSE^d^ score			
25–27	12 (6.5)	2 (4.9)	0.971
28–30	172 (93.5)	39 (95.1)	

Data are expressed as frequency (prevalence in %) or mean ± standard deviation or median

^a^*MAC* monitored anesthesia care group

^b^*AAA* asleep-awake-asleep group

^c^*KPS* Karnofsky score

^d^*MMSE* minimum mental state examination

### Intraoperative measures

A tidy flow chart for intraoperative measures in AAA and MAC groups was shown in Sup. Figure 2. Monitoring was carried out routinely for all the patients after entering the operating room. During the pre-awake stage, in the AAA group, after successful induction of anesthesia, placement of laryngeal mask (LMA) was executed. At the same time, mechanical ventilation was installed with a tidal volume of 6–8 ml/kg and a ratio of inhalation to exhalation of 1:2. In addition, the respiratory rate, end-tidal carbon dioxide partial pressure (PETCO2), and BIS value were maintained at a range of 10–14 times/min, 25–35 mmHg, and 40–60, respectively. In the MAC group, oxygen uptake was through a nasal catheter; meanwhile, an end-expiratory CO2 sampling tube was placed in the nostril to connect to the anesthesia machine. PETCO2, saturation of peripheral oxygen (SPO2), respiratory mobility, and thoracic fluctuation were dynamically monitored. In both groups, scalp nerves including supraorbital, auriculotemporal, greater occipital, and smaller occipital nerve were blocked, and local infiltration anesthesia was performed at the pin and incision sites. The drug regimen for nerve block and local infiltration anesthesia is as follows: 2% lidocaine (30 ml), 0.75% levobupivacaine (10 ml), 0.9% sodium chloride solution (20 ml), and 0.001% epinephrine (3 ml).

During the awake stage, infiltration anesthesia of dura was administered with 2% lidocaine (5–10 ml) for 10 min before opening the dura. In the AAA group, the plasma target concentration of propofol and remifentanil was gradually decreased after the removal of the bone flap. The patients were tried to wake up until the plasma effect chamber concentration of propofol reached 1 µg/ml and the BIS value reached above 80. The LMA was removed according to the patients’ compliance with the instructions prior to dural opening, and the infusion of propofol and remifentanil was discontinued. In the MAC group, the infusion of dexmedetomidine and remifentanil was lowered or intermittent based on OAA/S score to keep the patient in an arousal state. Cerebral cortical mapping was not performed before tumor resection until patients in both groups were in arousal state and not receiving intravenous anesthesia.

**Fig. 1:**
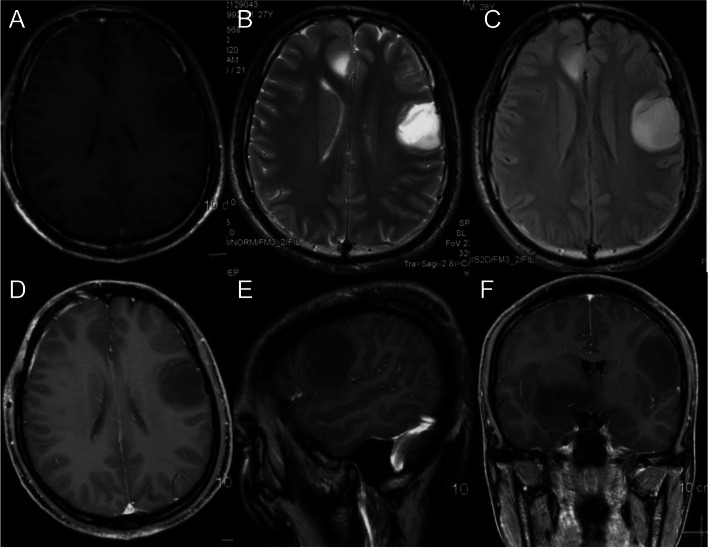
Preoperative magnetic resonance images of diffuse low-grade glioma in the left inferior frontal gyrus. **A** Axial T1. **B** Axial T2. **C** Axial T2 fluid-attenuated inversion recovery (FLAIR). **D**–**F** Axial, sagittal, and coronal T1 contrast

**Fig. 2:**
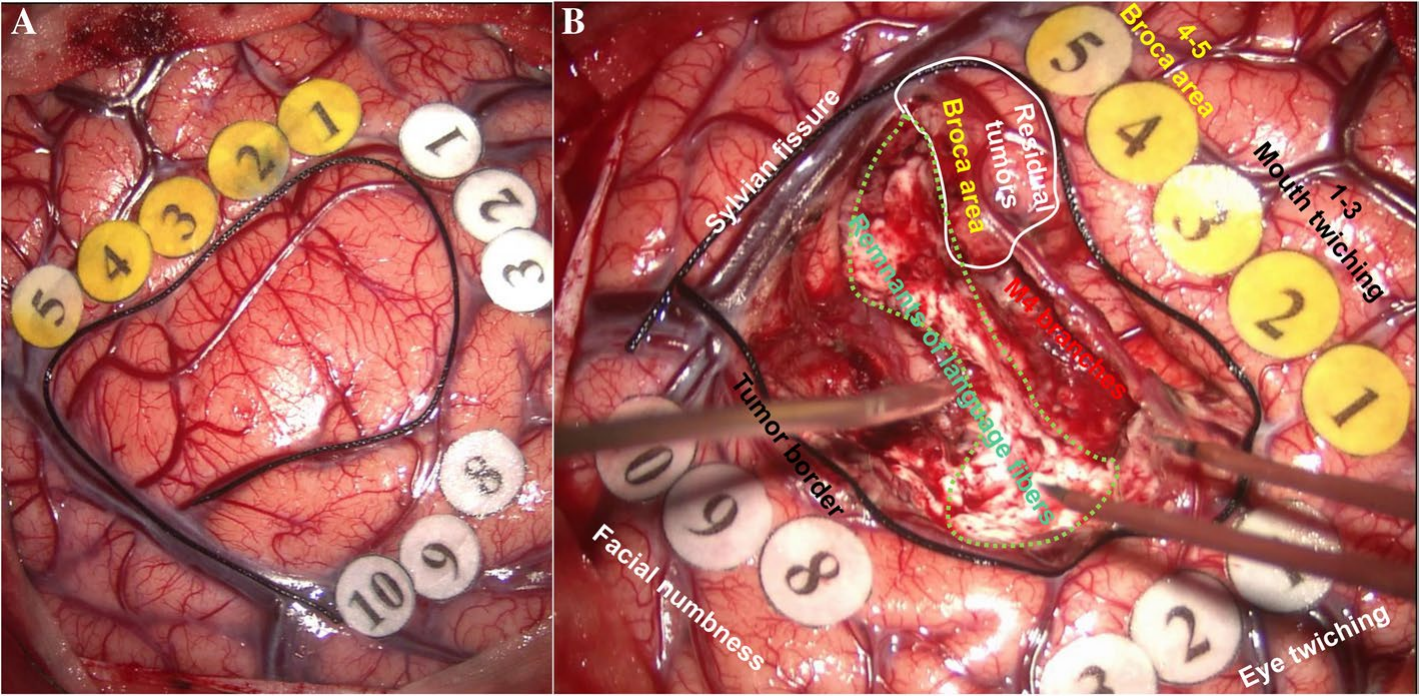
Intraoperative view of the cortex exposed by the left frontal–temporal craniotomy and brain mapping after stimulation and localization of eloquent sites. Right facial numbness is induced in stimulating the dorsal and caudal cortex of the tumors which is labeled with white 8–10, right mouth and eye involuntary convulsions were induced in stimulating the superior and anterior cortex of the tumors labeled with white 1–3 and yellow 1–3, and speech arrest was induced when brain cortex labeled with white and yellow 1–3 was stimulated. **A** Brain mapping before operation. **B** Tumor cavity after subtotal glioma resection

**Fig. 3:**
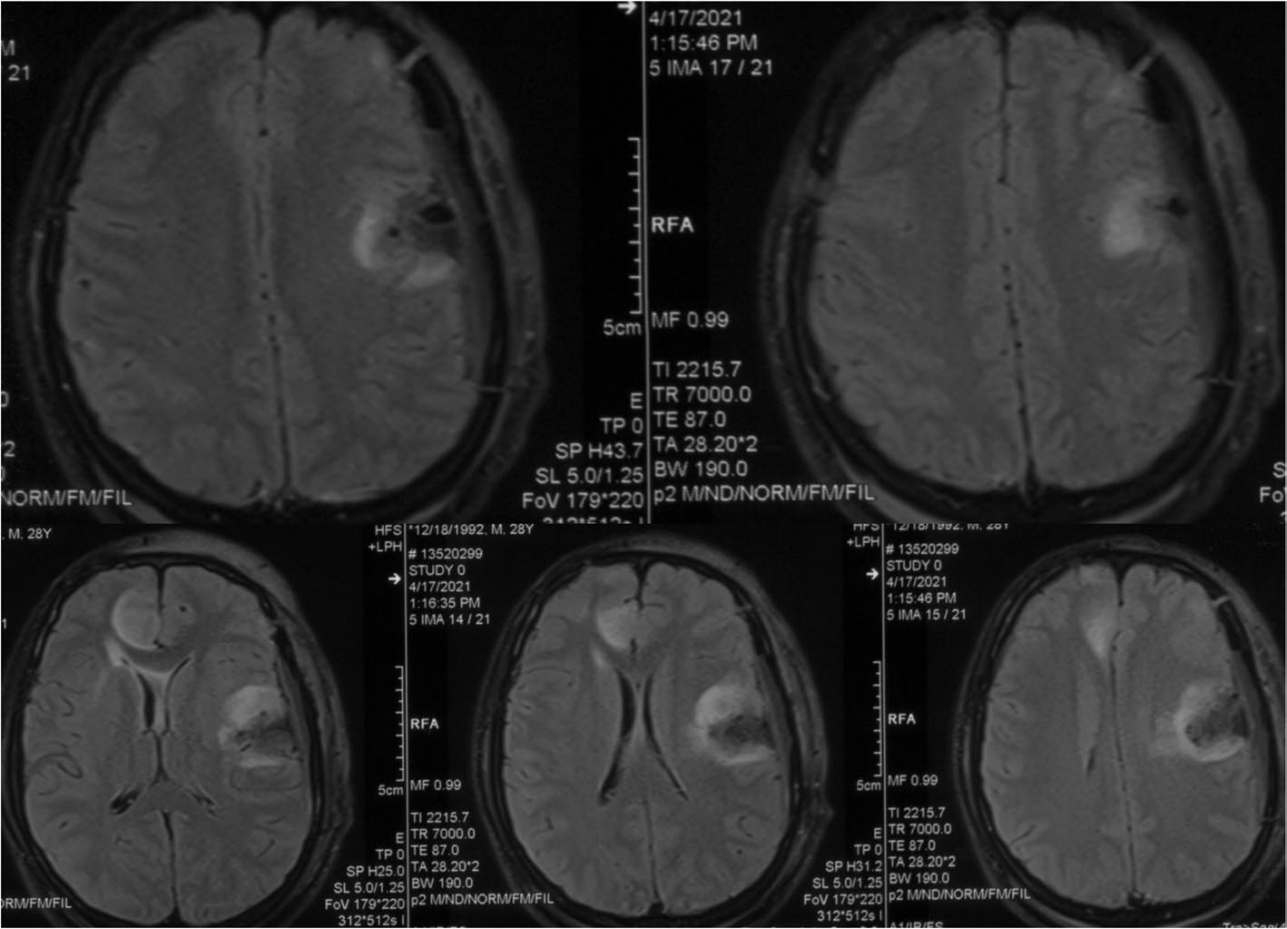
Postoperative magnetic resonance image (axial T2 fluid-attenuated inversion recovery)

When the residual tumor is close to the functional area, the tumor resection would not stop until the brain function is affected which was observed by cortical electrode monitors or communication with the patient with the purpose of maximal removal of the tumor but minimal brain damage. For example, a tumor located in left inferior frontal gyrus with language function was resected according to the principles mentioned above (Figs. 1, 2 and 3).

During the post-awake stage, after resection of lesions, both groups were treated with the method of MAC, and the intravenous anesthetic infusion speed was adjusted to maintain BIS value between 60 and 80 until the end of surgery. The intraoperative medication for complications is shown in the Sup. Table 1.

### Postoperative evaluation

In the present study, pre- and postoperative neurological function was assessed and recorded 1, 3, 5, and 7 days after surgery. The patient’s motor and language dysfunction was judged by at least two neurosurgeons based on the physical examination of the nervous system. The language was assessed with the Western Aphasia Battery (WAB) [7], and Fugl-Meyer Assessment (FMA) of sensorimotor recovery was performed for motor and sensory assessment by an experienced professional rater because its reliability and validity are well validated [8, 9] For follow-up, at 1, 3, and 6 months after operation, patients’ neurological function was assessed through Modified Rankin Scale (MRS).

During operation, speech assessment was performed with the help of a set of different images in a picture book including object naming, verb-generation exercises, counting, reading, monitoring of visual fields, spatial perception, and mathematics. And motor assessment was performed by a neurosurgical resident, and neurocognitive functions were performed by a neuropsychologist.

Postoperative MRI within 72 h was used to evaluate the extent of tumor resection which was interpreted by 2 neuroradiologists. Three-dimensional (3D) volumetric measurement of pre- and postoperative MRI was retrospectively conducted by 3D Slicer [10]. Manual segmentation was performed with the region-of-interest analysis to measure tumor volume based on T2 FLAIR-weighted images of low-grade glioma without contrast and T1-enhanced-weighted MR images of high-grade glioma with contrast. The extent of tumor resection was evaluated according to classification system by Sawaya [11], in which gross total resection (GTR) means tumor resection > 95%, subtotal resection (STR) 85–95%, and partial resection (PR) < 85%.

### Statistical analysis

Statistical analysis was conducted using SPSS (Statistical Package for Social Sciences, version 26.0). Quantitative data were presented as mean ± standard deviation, and count data were expressed in numbers (%). Homogeneity of variances was analyzed by Levene’s test. Student’s *t*-test or Mann–Whitney tests were used to compare quantitative parameters as applicable. Pearson, Fisher’s exact, and Yate’s chi-square tests were used for dichotomous criteria. Non-dichotomous counting data were analyzed by Kruskal–Wallis test. Since patients were gradually entered in the experiment during the process of admission, random number and blinding methods were not adopted. A *P*-value < 0.05 was considered significant for all tests.

## Results

### Demographic, tumor characteristics, and pathological characteristics

All operations were performed by the same group of neurosurgeons. The baseline data of patients were retrospectively analyzed in MAC group and AAA group, and it was found that there was no significant difference between the two groups in gender, KPS score, MMSE score, tumor grade, tumor growth site, and length of hospital stay. However, the mean age of patients in the MAC group was higher than that in the AAA group (48.26 ± 13.30 vs 39.41 ± 12.27; *t* = 3.903, *P* = 0.000). In addition, there was no significant difference in IDH mutation, MGMT methylation, Ki67 proliferation index, and 1p19q deletion between the two groups by pathological reports (Tables 1, 2 and 3).

**Table 2:** Grade and location of tumors

Preoperative characteristics	MAC^a^ (%)	AAA^b^ (%)	*p*-value
Grade			
I	14 (7.6)	0 (0.0)	0.692
II	58 (31.5)	17 (41.5)	
III	37 (20.1)	11 (26.8)	
IV	75 (40.8)	13 (31.7)	
Tumor location			
Frontal lobe	54 (29.3)	14 (34.1)	0.053
Temporal lobe	25 (13.6)	3 (7.3)	
Parietal lobe	18 (9.8)	4 (9.8)	
Occipital lobe	3 (1.6)	1 (2.4)	
Insular lobe	2 (1.1)	1 (2.4)	
Thalamus and basal ganglia	9 (4.9)	2 (4.9)	
Two or more brain lobes	46 (25.0)	16 (39.0)	
Multiple locations invading basal ganglia, cerebrum, or ventricle	27 (14.7)	0 (0.0)	

*GA* general anesthesia

*MAC* monitored anesthesia care group

*AAA* asleep-awake-asleep group

**Table 3:** Molecular pathological characteristics in two groups

	MAC (%)	AAA (%)	*p*-value
IDH mutation			
Yes	56 (30.4)	7 (17.1)	0.795
No	77 (41.8)	11 (26.8)	
Lost to follow-up	51 (27.7)	23 (56.1)	
MGMT promoter methylation			
Yes	46 (25.0)	14 (34.1)	0.553
No	83 (45.1)	20 (48.8)	
Lost to follow-up	55 (29.9)	7 (17.1)	
ki67 proliferation index	23.46 ± 21.34 165 (89.7)	24.38 ± 19.91 34 (82.9)	0.817
Lost to follow-up	19 (10.3)	7 (17.1)	
Deficient for both 1p and 19q			
Yes	28 (15.2)	1 (2.4)	0.392
No	81 (44.0)	10 (24.4)	
Lost to follow-up	75 (40.8)	30 (73.2)	

*MAC* monitored anesthesia care group

*AAA* asleep-awake-asleep group

### Intraoperative respiratory values, hemodynamic values, operation time, and rate of change to GA

During anesthesia, there was no significant difference in ASA score between the two groups. And blood gas analysis showed that there was no difference in lactic acid (Lac) between the two groups. The concentrations of PetCO2 (*P* = 0.000), PaCO2 (*P* = 0.000), and Glu (*P* = 0.003) were higher, while the SPO2 (*P* = 0.002) and PaO2 (*P* = 0.000) were lower in MAC group than those in AAA group (Table 4). When compared of the vital sign parameters between the two groups during anesthesia, it was shown that there was no significant difference in systolic blood pressure, diastolic blood pressure, heart rate, and pulse between the two groups. In terms of anesthesia of operation, there was no significant difference in anesthesia time, fluid inflow, output, and bleeding between the two groups. The operating time of MAC group was significantly less than that of AAA group (*P* = 0.039). The probability of conversion to general anesthesia (GA) of 9.8% in AAA group was significantly higher than that of 1.6% in MAC group (*P* = 0.027) (Tables 4 and 5).

**Table 4:** Intraoperative respiratory values

	MAC	AAA	*p*-value
Weight, kg	62.58 ± 9.45	63.57 ± 10.10	0.547
ASA^a^ score	2.07 ± 0.25	2.15 ± 0.36	0.174
SPO2, %	98.22 ± 2.00	99.24 ± 1.58	0.002
PetCO2, %	39.49 ± 3.12	37.15 ± 2.96	0.000
Lac^b^, mmol/L	1.41 ± 0.54	1.43 ± 0.88	0.924
Glu^c^, mmol/L	6.35 ± 1.40	5.45 ± 1.05	0.000
PaO2, mmHg	103.66 ± 19.45	113.98 ± 17.56	0.002
PaCO2, mmHg	45.87 ± 4.98	38.60 ± 6.68	0.000

^a^*ASA* American Society of Anesthesiologists

^b^*LAC* blood lactate level

^c^*Glu* blood glucose level

**Table 5:** Intraoperative hemodynamic values, operation time, rate of change to GA, and hospital stay

Intraoperative indicators	MAC	AAA	*p*-value
Systolic pressure, mmHg	127.07 ± 17.23	123.66 ± 15.19	0.244
Diastolic pressure, mmHg	78.30 ± 9.50	79.56 ± 10.57	0.454
Heart rate, times/min	76.81 ± 14.30	79.00 ± 18.98	0.490
Fluids infuse, ml	3268.06 ± 980.01	3110.24 ± 812.29	0.338
Fluids output, ml	2418.21 ± 758.37	2669.51 ± 766.23	0.057
Operative time, h	3.46 ± 1.26	3.90 ± 1.01	0.039
Anesthesia time, h	4.74 ± 1.35	4.85 ± 1.18	0.630
Intraoperative blood loss, ml	173.70 ± 101.46	179.27 ± 75.81	0.741
Rate of change to GA	3 (1.6)	4 (9.8)	0.027
Hospital stay, days	11.18 ± 5.43 days	11.27 ± 5.03 days	0.928

*MAC* monitored anesthesia care group

*AAA* asleep-awake-asleep group

### Intraoperative anesthesia-related drug usage

There was no significant difference in the dosage of local anesthetics between the two groups. Less remifentanil was used in the MAC group (*P* = 0.000), but more dexmedetomidine was used (*P* = 0.013) in AAA group. In terms of preventive medication, a higher utilization rate of antiemetic drugs (149 (81%) vs 25 (61%), *P* = 0.006) and vasoactive drugs (83 (45.1%) vs 26 (63.4%), *P* = 0.034) was found in MAC group (Table 6).

**Table 6:** Intraoperative anesthesia-related drug usage

	MAC (*n* = 184)	AAA (*n* = 41)	*p*-value
Local anesthetic dosage, µg	47.43 ± 8.17	49.41 ± 7.23	0.127
Remifentanil dosage, µg	711.06 ± 269.57	1079.44 ± 345.07	0.000
Dexmedetomidine dosage, µg	132.93 ± 96.77	94.82 ± 29.32	0.013
Proportion of patients using antiemetic drugs	149 (81.0)	25 (61.0)	0.006
Proportion of patients using vasoactive drugs	83 (45.1)	26 (63.4)	0.034

*MAC* monitored anesthesia care group

*AAA* asleep-awake-asleep group

### Extent of surgical resection and postoperative dysfunction

It seems that the extent of tumor resection in MAC group is higher than that in AAA group, but there was no significance (*P* = 0.076) (Table 7). We followed up the changes of motor, sensory, and language dysfunction of patients during 7 days after operation. It was found that the functional recovery of patients in MAC group was significantly better than those in AAA group on the 1st, 3rd, 5th, and 7th days after operation (*P* = 0.000). However, in the long-term follow-up, unfortunately, no differences in functional recovery were observed between the two groups (*P* = 0.327) (Tables 8 and 9).

**Table 7:** Extent of surgical resection

Extent	MAC (%)	AAA^b^ (%)	*p*-value
Total resection	117 (63.6)	19 (46.3)	0.076
Subtotal resection	52 (28.3)	15 (36.6)	
Partial resection	15 (8.1)	7 (17.1)	

*MAC* monitored anesthesia care group

*AAA* asleep-awake-asleep group

**Table 8:** Postoperative short-term dysfunction

	MAC (*n* = 184)	AAA (*n* = 41)	*p*-value
Postoperative day 1			0.003
Impairment of language function	6 (3.3)	6 (14.6)	
Impairment of motor function	36 (19.6)	16 (39.0)	
Impairment of both motor and language function	4 (2.2)	0	
Sensory disturbance	4 (2.2)	0	
Coma	1 (0.5)	0	
Postoperative day 3			0.000
Impairment of language function	14 (7.6)	10 (24.4)	
Impairment of motor function	34 (18.5)	18 (43.9)	
Impairment of both motor and language function	4 (2.2)	0 (0.0)	
Sensory disturbance	4 (2.2)	0 (0.0)	
Coma	1 (0.5)	0	
Postoperative day 5			0.000
Impairment of language function	8 (4.3)	10 (24.4)	
Impairment of motor function	36 (19.6)	16 (39.0)	
Impairment of both motor and language function	4 (2.2)	0 (0.0)	
Sensory disturbance	4 (2.2)	0 (0.0)	
Coma	1 (0.5)	0	
Postoperative day 7			0.000
Impairment of language function	4 (2.2)	6 (14.6)	
Impairment of motor function	34 (18.5)	18 (43.9)	
Impairment of both motor and language function	4 (2.2)	0 (0.0)	
Sensory disturbance	4 (2.2)	0 (0.0)	
Coma	1 (0.5)	0

**Table 9:** Postoperative long-term functional status

	Number of follow-up/ all patients (%)	Postoperative long-term functional status			*p*-value
		(MRS 0–1) (%)	(MRS 2–3) (%)	(MRS 4–5) (%)	
MAC	175/184 (95.1)	135 (77.1)	37 (21.2)	3 (1.7)	0.327
AAA	34/41 (82.9)	25 (73.5)	7 (20.6)	2 (5.9)	

*MAC* monitored anesthesia care group

*AAA* asleep-awake-asleep group

## Discussion

It is shown in the present study that the incidence of conversion to GA and operative time was lower in the MCA group than that in the AAA group. Early functional recovery of patients in MAC group was significantly better than those in AAA group on the 1st, 3rd, 5th, and 7th days after operation. Intraoperative concentrations of PetCO2, PaCO2, and Glu were significantly higher, while SPO2 and PaO2 were significantly lower in MAC group than those in AAA group. Less remifentanil but more dexmedetomidine, antiemetic, and vasoactive drugs were used in the MAC group.

AC is a demanding but safe procedure for brain tumors within or adjacent eloquent areas. According to a European Low-Grade Glioma Network survey [6], eighteen centers (90%) preferably used either MAC or AAA; only three centers reported AAA and MAC simultaneously [12–14]. In fact, there were few studies comparing the advantages or disadvantages of these two anesthetic techniques [13–17], especially when multiple monitoring was in use. A meta-analysis only made comparisons among AC failures, conversion to GA, intraoperative seizures, and neurological dysfunction, but those data was from many published studies which were performed with either MCA or AAA approaches. Three studies are retrospectively comparing the MCA and AAA technique performed by a single center or a single neurosurgeon like our study, in which one study [14] concluded that the AAA technique may provide better results with respect to agitation and seizure, and another study [13] suggested a similar perioperative outcome between the two techniques, with shorter operative time for MAC, and the third study demonstrated that MCA with sole dexmedetomidine reduces respiratory and cardiovascular adverse events with a low need for antihypertensive and vasoactive drugs, probably ensuring more rapid surgery and reducing length of hospitalization [12]. Our mean operative time was 3.46 ± 1.26 h in MAC group while 3.90 ± 1.01 h in AAA group; even if we became more and more familiar with the whole procedure in operating room, there has been only a slight reduction in our surgical time in recent years. Consistent with Eseonu’s study [13], the operative time of MCA group was about 27 min shorter than that of AAA group. The reasons for the shorter operative time may be as follows. First, intubation and extubation of LMA in pre-awake stage prolonged the operation [18]; however, we did not intubate LMA again after tumor resection consistent with Olsen’s study [19]. Second, we have to waste some time waiting for the patient to be fully conscious before the LMA is removed safely in AAA group, although anesthesiologist can reduce the amount of anesthetic to wake up the patient in advance [18]. In addition, the shorter operative time in MAC group may indicate less cost in America [20], which was certified by Eseonu [13] who suggested a better cost effectiveness with MCA technique in the patients undergoing AC. However, we found no difference in hospital stay and costs between the two anesthetic techniques.

In our study, not only the proportion of conversion to GA was significantly lower in MCA group than that in AAA group but also the whole proportion of conversion (0.03%, 7 in 225 patients) is much lower than that reported of 1–2% which is consistent with the outcomes of Stevanovic’s meta-analysis [15], in which the odds ratio (OR) comparing AAA to MAC was 2.17 and the likelihood ratio test (LR test) showed a significant *P*-value of 0.03. This may belong to our team of experienced professional anesthetists. The reasons for conversion to GA include LMA leakage, respiratory insufficiency, intraoperative bleeding, pain, brain bulge, seizure, severe restlessness, and acute brain edema [15]. However, intraoperative seizures, which is one of the most common cause of failure in AC [21], are also one of the causes for conversion to GA in our study. Even though some patients need blood transfusion, intraoperative bleeding can be effectively controlled without the need to switch to GA.

The incidence of airway- and ventilation-related complications in AC has been reported to be 1.8 to 18% in the literature [22–24]. In the study with 18% airway- and ventilation-related complications, 4 (9%) patients developed decreases in respiratory rate and oxygen saturation (90%), and all these patients recovered by a brief period of a short period of manual jaw thrust or a short application of oxygen and ventilation by mask [24]. In our study, desaturations or airway obstruction was not observed, possibly because of good oxygenation via the nasal trumpet prior to sedation, real-time monitoring of PetCO2, timely adjustment of drug dosage, less usage of remifentanil, and more dexmedetomidine which is believed to be associated with fewer respiratory adverse events compared with propofol and remifentanil during AC for supratentorial tumor resection [25]. Navdeep’s study [22] also demonstrated that the episodes of airway obstruction leading to desaturation and hypertension were more in propofol group as compared to dexmedetomidine. Although our study demonstrated that PetCO2, PaCO2, and Glu levels were significantly higher in the MCA group than that in the AAA group, the incidence of brain edema was not observed in the two groups except for patient suffering from generalized seizures.

Intra- and postoperative nausea and vomiting have to be stopped for AC because these nausea and vomiting may contribute to inadvertent brain swelling and enhanced risk of aspiration, discomfort, and distress. The incidence of intra- and postoperative nausea and vomiting is between 0 and 30% [15, 24], while our incidence is 2.17% in MAC group and 0 in AAA group. This low incidence may be due to the use of pre- and postoperative intravenous antiemetics when necessary [26].

Although the pulse pressure difference in MAC group was greater than that in AAA group, there was no significant difference in systolic blood pressure, diastolic blood pressure, heart rate, and pulse between MAC and AAA group. This result is coincident with Eseonu’s study that intraoperative hypertension occurs equally in 8% of MAC and 9.7% of AAA but different from Dilmen’s study [13, 14] in which Dilmen et al. demonstrated that blood pressures were significantly higher in the MCA group during pinning, and heart rate and blood pressures were significantly higher in the MCA group than that in the AAA group during the skin incision. The incidence of intraoperative hypertension in this study occurred 22.82% in MAC and 17.07% in AAA group. This was coincident with the reported incidence of AC intraoperative hypertension that ranges from 16.7 to 24% [22, 27], but the incidence of intraoperative hypertension is higher than Eseonu’s study [13].

The principle of modern glioma surgery is to remove the tumor safely and maximally and improve symptom management, quality of life, progression-free survival (PFS), and prognosis in both low- and high-grade glioma. AC, known to preserve the quality of life in patients with low-grade glioma, is also able to significantly increase the extent of resection for lesions located in functional regions [4, 28]. Intraoperative application of electrophysiological monitoring, fluoroscopy, and intraoperative magnetic resonance helps to increase the advantages of AC to resect the tumor safely and maximally [29–31] because supertotal resection may provide survival benefits in HGG. In a large study reviewing 1229 patients with GBM over 19 years, prolonged survival was seen in patients that underwent greater than 53% resection of the T2/FLAIR abnormality in addition to GTR of the 259 contrast-enhancing region (20.7 vs. 15.5 months, *p* < 0.001) and low-grade gliomas [32]. In our study, the extent of tumor resection was compared for the first time between MAC and AAA groups, and it was found that there was no significant difference of the extent of tumor resection between the two groups, but patients in the MAC group seemed to be more likely to undergo total resection. It is suspected that there are more patients converted to GA because of generalized seizures in AAA group which is in fact equal to general anesthesia. The neurosurgeons’ mood may also have influence on the manipulation of tumor removal. It is believed the intervention of AAA techniques during operation may result in bad temper of the surgeons.

In order to follow the principle of modern glioma surgery, new techniques such as neuro-navigation, brain-mapping, and brain-monitoring techniques were applied during our tumor resection. Besides, functional boundaries have to be beard in mind constantly. With the help of these techniques, we have to locate the tumor boundaries and then resect the tumors inside the functional boundaries. Normally after we approach the functional boundary by 1 cm with the help of cortical and subcortical electrical stimulation, communication was carried on continuously by a neurologist and speech pathologists simultaneously with the awake patient. The surgery keeps on and does not stop until the onset of neurologic dysfunction of the patient occurred like aphasia or paralysis. In our study, early functional recovery of patients in MAC group was significantly better than those in AAA group on the 1st, 3rd, 5th, and 7th days after operation. The reasons for this difference may be explained as follows: first, there are more patients converted to GA because of generalized seizures in AAA group which is equivalent to general anesthesia; second, it may be related to the better cooperation of doctors with patients in MAC group, while in AAA group, cooperation is usually hard to proceed between the doctors and the just arousal lethargy patients.

The limitations of study are that the study is a retrospective one. Even though demographic characteristics were consistent in group AAA and MAC, AAA management in glioma awake craniotomy was used in the early stage of our clinical practice since 2009, and we started using MAC since 2013. That is why the sample was different between the two groups. So, the potential statistical bias in this two groups may exist. In addition, our study was conducted in a single institution. Therefore, large randomized controlled trials are necessary to further evaluate the benefits of the two techniques.

## Conclusion

A successful AC requires the cooperation of a multidisciplinary team, a good understanding of airway management and sedation protocols, and the ability to manage adverse intraoperative issues. Although both the MAC and AAA techniques could provide safe and effective anesthesia management for an AC for brain gliomas resection in eloquent areas, it seems that MAC has more advantages in the management for resection of gliomas in eloquent brain areas, and MAC combined with multiple monitoring such as cerebral cortical mapping neuronavigation and ultrasonic detection is worthy of popularization for the resection of gliomas in eloquent brain areas.

## Supplementary Information

**Additional file 1: Sup. Fig. 1. **A flow chart for illustration of groups.

**Additional file 2: Sup. Fig. 2. **A tidy flow chart for intraoperative measures in AAA and MAC groups.

**Additional file 3: Sup. Table 1. **Intraoperative medication for complications.** Sup. Table 2. **Modified Rankin Scale.

## Data Availability

Materials described in the manuscript, including all relevant raw data, will be freely available to any scientist wishing to use them for noncommercial purposes, without breaching participant confidentiality.
