# Supplementary figures and images for: Monitored anesthesia care and asleep-awake-asleep techniques combined with multiple monitoring for resection of gliomas in eloquent brain areas: a retrospective analysis of 225 patients
Source: Chin Neurosurg J. 2022 Dec 30;8:45. doi: 10.1186/s41016-022-00311-2 (PMC9801549; doi:10.1186/s41016-022-00311-2)

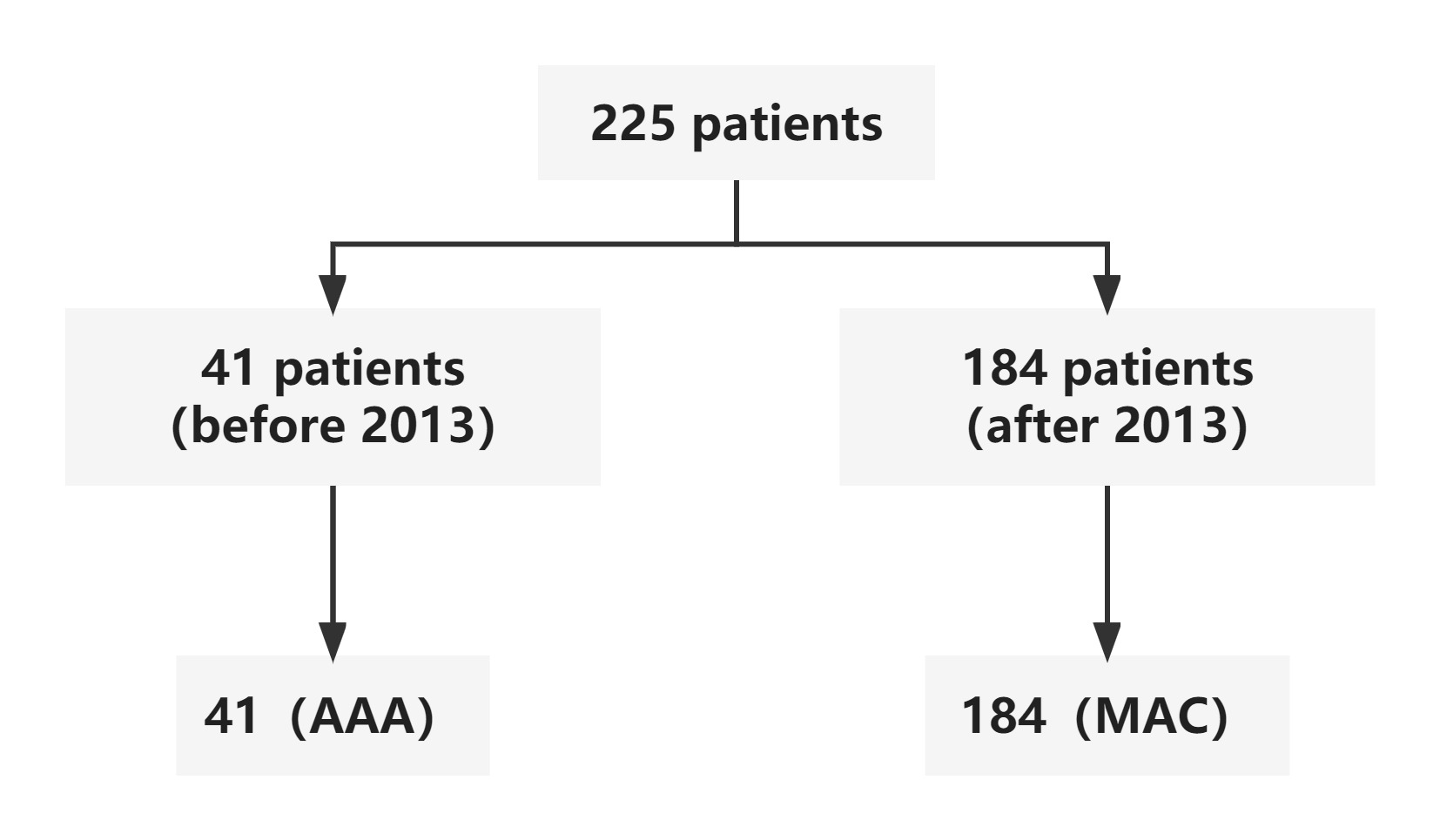

Supplement: Supplementary file 1 — Additional file 1: Sup. Fig. 1. A flow chart for illustration of groups. [file 41016_2022_311_MOESM1_ESM.jpg]

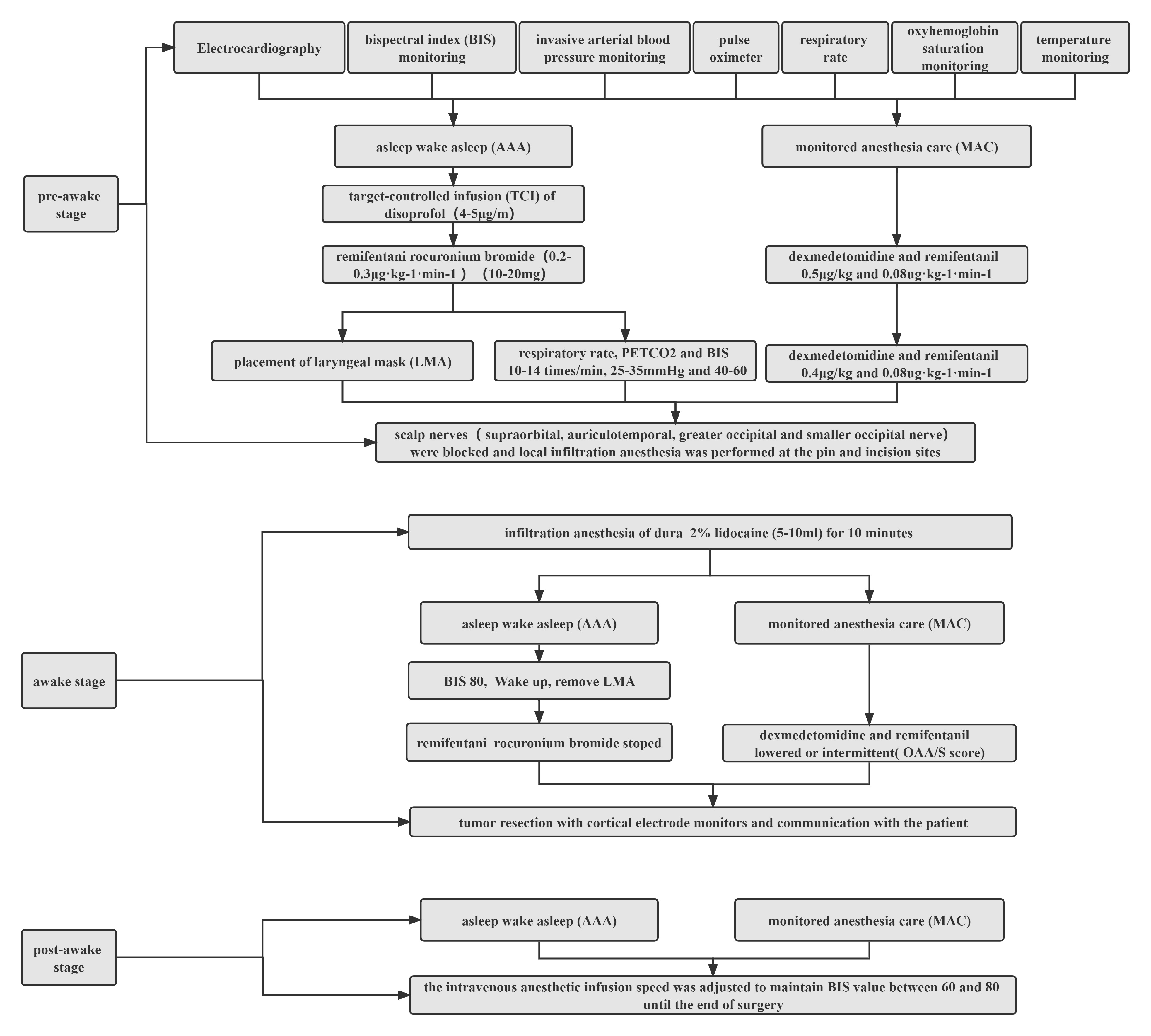

Supplement: Supplementary file 2 — Additional file 2: Sup. Fig. 2. A tidy flow chart for intraoperative measures in AAA and MAC groups. [file 41016_2022_311_MOESM2_ESM.jpg]
