# Supplementary material for: Monitored anesthesia care and asleep-awake-asleep techniques combined with multiple monitoring for resection of gliomas in eloquent brain areas: a retrospective analysis of 225 patients
Source: Chin Neurosurg J. 2022 Dec 30;8:45. doi: 10.1186/s41016-022-00311-2 (PMC9801549; doi:10.1186/s41016-022-00311-2)
Supplement: Supplementary file 3 — Additional file 3: Sup. Table 1. Intraoperative medication for complications. Sup. Table 2. Modified Rankin Scale. [file 41016_2022_311_MOESM3_ESM.docx]

sup. table1 Intraoperative medication for complications

| complication | Therapeutic regimen(dose) |
| --- | --- |
| Pain or discomfort | Fentanyl (25-100μg/times) |
| High blood pressure | Nicardipine (0.2-0.4mg/times) |
| Low blood pressure | Ephedrine (5-10mg/times) |
| Heart rate<50times/min | Atropine (0.3-0.5mg) |
| Heart rate>100times/min | Esmolol (0.5mg/kg) |

sup. Table 2 Modified Rankin Scale

| Grade | Signs and symptoms |
| --- | --- |
| 0 | No symptoms |
| 1 | No notable disability despite symptoms: able to carry out all usual tasks and activities |
| 2 | Slight disability: unable to carry out all previous activities but able to look after own affairs without assistance |
| 3 | Moderate disability: requiring some help, but able to walk without assistance |
| 4 | Moderately severe disability: unable to walk without assistance, unable to attend to own bodily needs without assistance |
| 5 | Severe disability: bedridden, incontinent, and requiring constant nursing care and attention |
| 6 | Death |
